# Psychomotor regression due to vitamin B12 deficiency

**DOI:** 10.11604/pamj.2018.30.152.12046

**Published:** 2018-06-20

**Authors:** Amal Bousselamti, Brahim El Hasbaoui, Hanae Echahdi, Yamna Krouile

**Affiliations:** 1 Department of Paediatrics 2, Unit of Endocrinology and Neuropediatrics, Children’s Hospital, Faculty of Medicine and Pharmacy, University Mohammed V, Rabat, Morocco

**Keywords:** Vitamin B12 deficiency, breastfeeding, psychomotor regression, hypotonia, hyperhomocysteinemia

## Abstract

A vitamin B12 deficiency in infants is rare, but may sometimes be seen in breastfed babies of strict vegetarian mothers. Vitamin B12, also known as cobalamin, is only found in meat and other animal products. Most babies have a sufficient supply as long as the mother was not deficient herself. Symptoms and signs of vitamin B12 deficiency appear between the ages of 2 to 12 months and include vomiting, lethargy, failure to thrive, hypotonia, and arrest or regression of developmental skills. Urinary concentrations of methylmalonic acid and homocystine are characteristically elevated in vitamin B12 deficiency. Early treatment for a vitamin B12 deficiency in an infant involves immediate administration of vitamin B12 to the baby and the breastfeeding mother. The infant and mother will each receive an injection of vitamin B12 containing 1,000 mcg or more of the vitamin, and the mother will continue to receive injections every month to raise her own stores. After the initial injection, the baby will often receive future vitamin B12 through food sources. We present a case of vitamin B12 deficiency in a 9-month-old girl presented with psychomotor regression, hypotonia and lethargy. The child was exclusively breast-fed from birth by a mother who was on strict vegetarian diet and belong to a low socio-economic status. Laboratory data revealed bicytopenia with macrocytic anemia and methylmalonic acid in the urine, consistent with vitamin B12 deficient anemia. The Brain CT revealed a cerebral atrophy and delayed myelination. Vitamin B12 supply was effective on anaemia and psychomotor delay. This case figures out the importance of an early diagnosis in front of psychomoteur regression and hypotonia, given the risk of incomplete neurologic recovery due to vitamin B12 deficiency mainly in the setting of maternal nutritional deficiency.

Vitamin B12 deficiency (<148 pmol/L) is a major public health problem worldwide [1]. Maternal vitamin B12 deficiency has been associated with increased risk of common pregnancy complications [2] and impaired growth and brain development in offspring [3]. Maternal and fetal vitamin B12 concentrations are thought to be closely associated throughout pregnancy [4]. Previous cross sectional studies in Norway, Turkey, and Brazil have noted correlations between maternal and infant vitamin B12 status at delivery [5]. In developed countries, manifestation is restricted to breast-fed infants of either vegetarian or vegan mothers or mothers with pre-existing malabsorption [6], and to rare cases of genetic defects of vitamin B12 metabolism [7]. Vitamin B12 is found primarily in animal source foods and functions as essential cofactor for methylation of homocysteine to methionine and conversion of methylmalonyl-CoA [7]. Adult hepatic stores of 1-4 mg balance a vitamin B12 devoid diet for several years. In contrast, infantile vitamin B12 body stores comprise about 25µg and may be much lower, if the infant's mother is vitamin B12 deficient [7]. The clinical features of vitamin B12 deficiency include hematologic and neurologic alterations. Hematologically, in about 70% megaloblastic anemia is present. Neurological symptoms comprise muscle weakness, paresthesia and atactic paraplegia in adults. Typical neurological symptoms in infancy are irritability, weakness, developmental delay and failure to thrive, finally apathy and coma [8].

The biochemical mechanisms underlying the neurological symptoms of vitamin B12 deficiency are poorly understood. Impaired myelination, demyelination, axonal degeneration and general cerebral atrophy display morphological correlates [8]. Altered myelin formation and integrity due to dysregulated incorporation of fatty acids have been hypothesized as well as an accumulation of lactate or neurotoxic cytokines [8, 9]. With myelination being most active in the first 6 months of life, infantile vitamin B12 deficiency may cause substantial neurological damage [8]. Initial improvement upon therapy might not immediately affect morphological changes, but might be due to a resolution of neurotransmitter imbalance [8]. Thus, treatment does not necessarily result in appropriate psychomotor long-term development. We report a case of vitamin B12 deficiency in a 9-month-old girl presented with psychomotor regression, hypotonia and lethargy.

## Patient and observation

We report the 9-month-old exclusively breast-fed girl who had been referred due to progressive hyptonia and apathy. Pregnancy and birth history were unremarkable. The girl had been born full-term (39 weeks) with a birth weight (40^th^percentile), length (70^th^ percentile) and head circumference (80^th^ percentile) within the normal range. After normal development within the first 4 months, she had lost developmental milestones, activity had generally decreased, and she had progressively feeding difficulties. The girl was pale and tachycardic at presentation, motor skills and cognitive development of infants were delayed. There were no organomegaly and dysmorphic features. Neurological examination revealed apathy and profound hypotonia with brisk deep tendon reflexes. She had head control, however, axial and peripheral tonus had slightly weakened. Infants was exclusively breast-fed and received no vitamin supplementation. Blood count pronounced: hemoglobin (Hb) level at 9.2 g/dL; mean corpuscular volume (MCV), 114 fL; leukocyte count, 5300 cells/mm^3^; platelet count, 412 109 cells/mm^3^; blood chemistry and endocrine analysis were unremarkable including levels of lactate, pyruvate, ammonia, thyroidal parameters, prolactin and ACTH. Parvovirus B19, Hepatitis C and B, EBV and CMV infections were excluded.

**Figure 1:**
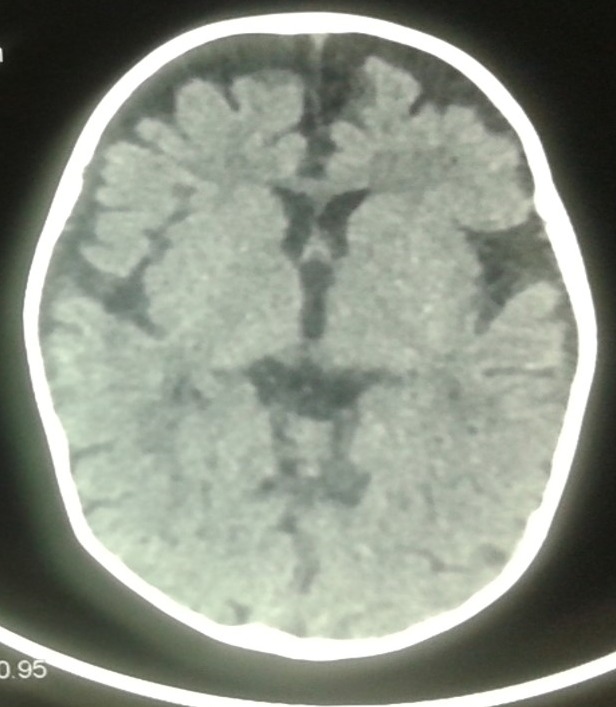
Brain CT revealed cerebral atrophy and delayed myelination

Because of macrocytic anemia and bicytopénia, bone marrow aspiration was realised and revealed dysplastic features consistent with reactive myelodysplasia. Serum folic acid level was normal while vitamin B12 level was less than 83pg/mL (normal range, 200-400); and serum homocystein level very high at 47,22UM/L (4.6-8.1). The brain CT revealed cerebral atrophy and delayed myelination (Figure 1). This metabolic analysis documented significantly diminished vitamin B12 serum levels as well as methylmalonic aciduria, low methionine and markedly elevated homocysteine serum levels indicating vitamin B12 deficiency. Following erythrocyte transfusion, supplementation of vitamin B12 was initiated days after admission, starting with intramuscular injections everyday 1mg for 1 week, then 1mg every week for 4 weeks, and then 1000 mg every month for 6 months. A follow-up 6 weeks after admission revealed normal serum vitamin B12 levels, normal urinary excretion of methylmalonic acid and hematologic parameters were normalized. Regarding neurocognitive development, the girl had achieved appropriate head control and was able to sit with slight assistance. Investigations on the mother of patient showed complete deficiency of vitamin B12 (<30 pg/ml) without macrocytic anemia, anti-parietal cell antibodies (APCA), Anti-intrinsic factor were not found. This mothers was on strict vegetarian diet (excluded all animal products from her diet) belong to a low socio-economic status explaining this vitamin B12 deficiency and she was also treated with intramuscular cyanocobalamin.

## Discussion

From one population to another and among age groups, the prevalence of vitamin B12 deficiency varies considerably. In the US, a prevalence of 1-3% has been reported in children less than 4 years [7-9], whereas a high prevalence of up to 40% has been described in children of developing countries due to malnutrition [10]. Vitamin B12 is found primarily in animal source foods and functions as essential cofactor for methylation of homocysteine to methionine and conversion of methylmalonyl-CoA [11]. Adult hepatic stores of 1-4 mg balance a vitamin B12 devoid diet for several years. In contrast, infantile vitamin B12 body stores comprise about 25 µg and may be much lower, if the infant's mother is vitamin B12 deficient [11]. Although the nutritional Vitamin B12 deficiency in infancy was already described in 1962 [10] and several times later e.g [12-14] and recently reviewed by Dror et al. [15] the general awareness is still not appropriate. Vitamin B12 deficiency in infants is mostly nutritional due to low levels of vitamin B12 in the milk of their Vitamin B12-deficient but still asymptomatic mothers. Inherited disturbances of Vitamin B12 metabolism are rare. Maternal Vitamin B12 deficiency may be caused by various gastrointestinal diseases including achlorhydria, Helicobacter pylori infection, celiac disease, Crohn disease, pancreatic insufficiency, treatment with proton pump inhibitors or by insufficient Vitamin B12 intake in vegetarian diet. Due to active placental Vitamin B12 transport in utero resulting in fetal serum Vitamin B12 levels twice those in maternal serum. Placental transporter proteins modulate nutrient transfer to the fetus during gestation [16]. Transcobalamin (TC) and haptocorrin are the primary transporters of vitamin B12; transcobalamin binds to over 70% of vitamin B12 transported across the placenta, compared to 10%-30% in maternal circulation [17]. Although it is known that the placenta produces TC, which may be released into the maternal and fetal circuit, the mechanisms and pathways of vitamin B12 transport from the maternal to the fetal circuit are largely unknown [18]. Few data exist on the ability of the placenta to modify vitamin B12 transport in response to maternal vitamin B12 status.

Pregnant adolescents, which represent over 5% of the U.S obstetric population [19], are at an even greater risk for micronutrient deficiencies and pregnancy complications [20]. Even children of Vitamin B12-deficient mothers usually have enough Vitamin B12 for adequate prenatal development. However, they are born with lower stores of Vitamin B1215 and following their depletion, clinical manifestation gradually develops. The mains factors influencing the speed and severity of manifestation of Vitamin B12 deficiency in infants are the severity of maternal Vitamin B12 deficiency, combined with the duration of deficiency. There is also a relation between time of diagnosis and the prognosis, Graham et al. [12] and Von Schenck et al. reported a good outcome in children diagnosed and supplemented with Vitamin B12 before the age of 10 months, and permanent neurological abnormalities in children diagnosed after 1 year of age. Vitamin B12 (cobalamin) has a variety of biological functions but above all it is essential for hematopoiesis and the development of nervous system. In adults it may present as megaloblastic anaemia, polyneuropathy, subacute combined neurodegeneration of spinal cord, dementia or depression. The clinical impairment of mature nervous system develops slowly in months or years [21]. This contrasts with Vitamin B12 deficiency in infants who undergo extensive growth and development of the brain: Vitamin B12 deficiency may cause severe impairment in only a few weeks. The most common symptoms include failure to thrive, hypotonia, irritability or lethargy, developmental delay and even regression, [22,23] epilepsy or movement disorder [13,14]. Brain atrophy, delayed myelination [24], polyneuropathy and abnormal evoked responses 9 were reported. Laboratory findings usually include macrocytic anaemia, bi or pancytopenia and metabolic analysis documented significantly diminished vitamin B12 serum levels as well as methylmalonic aciduria, low methionine and markedly elevated homocysteine serum levels indicating vitamin B12 deficiency while the bone marrow examination often shows megaloblastic. Vitamin B12 supplementation normalizes the hematological and metabolic disturbances, but early treatment is crucial to prevent neurological sequelae such as learning or behavioural problems, secondary epilepsy or mental retardation.

## Conclusion

Infantile vitamin B12 deficiency is a treatable. Maternal Vitamin B12 status is a major factor influencing the severity of Vitamin B12 deficiency in infants. Clinical presentation of severe deficiency was typical, consisting of failure to thrive, hypotonia, developmental delay/regression, microcephaly and megaloblastic anaemia. The occurrence of such symptoms in exclusively breastfed infant should necessitate the examination of blood vitamin B12. Early diagnosis and therapy seem to be crucial for the prevention of permanent neurological squeal. Our case illustrate that vitamin B12 deficiency should be considered in the differential diagnosis of psychomotor regression and hypotonia mainly in the setting of maternal nutritional deficiency.

## Competing interests

The authors declare no competing interests.
